# Gamma knife radiosurgery for a rare Rosette-forming glioneuronal tumor in the brainstem region: A case report and literature review

**DOI:** 10.1097/MD.0000000000041869

**Published:** 2025-03-14

**Authors:** Zhipeng Shen, Runzhu Ge, Dongxue Zhou, Yonglong Jin, Jie Wang, Shuyan Zhang, Chao Liu, Zishen Wang, Wei Wang, Yinuo Li, Weiwei Wang, Shosei Shimizu

**Affiliations:** a Department of Neurosurgery, Zhejiang University School of Medicine Children’s Hospital, Hangzhou, China; b National Clinical Research Center for Child Health, Hangzhou, China; c Graduate School, Chengde Medical College, Chengde, China; d Department of Pediatric Radiation Therapy Center/Pediatric Proton Beam Therapy Center, Hebei Yizhou Cancer Hospital, Zhuozhou, China; e Department of Radiotherapy, The Affiliated Hospital of Qingdao University, Qingdao, China; f School of Public Health, Qingdao University, Qingdao, China; g Department of Radiotherapy Physics and Technology, Hebei Yizhou Cancer Hospital, Zhuozhou, China; h Department of Radiology, Hebei Yizhou Cancer Hospital, Zhuozhou, China; i Department of Radiation Oncology, University of Tsukuba, Tsukuba, Ibaraki, Japan.

**Keywords:** brainstem tumor, case report, frameless fractionated radiosurgury, gamma knife radiosurgery, radiation therapy, Rosette-forming glioneuronal tumor

## Abstract

**Rationale:**

Rosette-forming glioneuronal tumor (RGNT) is a rare primary nervous system tumor, with limited treatment guidelines due to its rarity, especially in the brainstem. This report presents a unique case of brainstem RGNT treated with gamma knife radiosurgery (GKRS).

**Patient concerns:**

A 35-year-old woman sought medical attention after sudden syncope and rapid decline in consciousness. Magnetic resonance imaging revealed a mass in the pineal region, extending to the brainstem and thalamus. Due to the critical location, only partial resection of the pineal tumor was possible, leaving most of the residual tumor in the vital brainstem area, requiring urgent intervention to control its growth and prevent sudden complications.

**Diagnoses:**

Postoperative histopathological results confirmed a diagnosis of RGNT.

**Interventions:**

The patient underwent 25 Gy/5 fractions of GKRS using the frameless Gamma Knife ICON™ (Elekta) device, as confirmed by cone-beam computed tomography scans for precise dose distribution and patient alignment.

**Outcomes:**

GKRS was performed successfully and safely. The tumor significantly shrank 3 months post-GKRS, and the patient experienced symptom relief without any adverse effects.

**Lessons:**

GKRS is considered an effective modality for RGNT in high-risk brainstem areas, minimizing risks while controlling tumor growth and alleviating symptoms. In addition, the frameless Gamma Knife ICON™ device enhanced patient comfort and treatment precision. GKRS offers a noninvasive alternative for similar RGNT cases.

## 1. Introduction

The World Health Organization (WHO) first described the rosette-forming glioneuronal tumor (RGNT) of the fourth ventricle in the 2007 classification of central nervous system tumors. RGNT is generally considered to exhibit benign progression,^[1]^ predominantly affecting young adults with an average age of 33.^[2]^ Surgical resection has been the primary treatment with favorable outcomes, as studies show high progression-free survival and overall survival rates.^[3]^ However, in some cases, particularly in critical regions such as the thalamus and brainstem, recurrence or rapid progression can occur as surgery fails to completely remove it. One study indicated that among 91 cases of RGNT, 14% of the patients experienced recurrence or rapid progression/dissemination after surgical treatment.^[4]^ The absence of comprehensive prospective studies or standardized treatment guidelines globally further complicates the management of RGNT, particularly in high-risk areas.

Although benign histology of RGNT often suggests a positive prognosis, its location in vital areas such as the brainstem poses significant clinical challenges. The brainstem controls essential functions such as, breathing and motor control, and even a small tumor in this region can cause severe neurological deficits.^[5]^ For tumors in inoperable or critical locations, adjunctive therapies such as radiotherapy are considered, with gamma knife radiosurgery (GKRS) emerging as a key option. This case report highlights the urgent intervention required for a patient with RGNT in the brainstem, where GKRS was effectively utilized to manage the tumor and preserve neurological function using the latest GK-ICON™ device.

## 2. Case presentation

**Figure 1.**
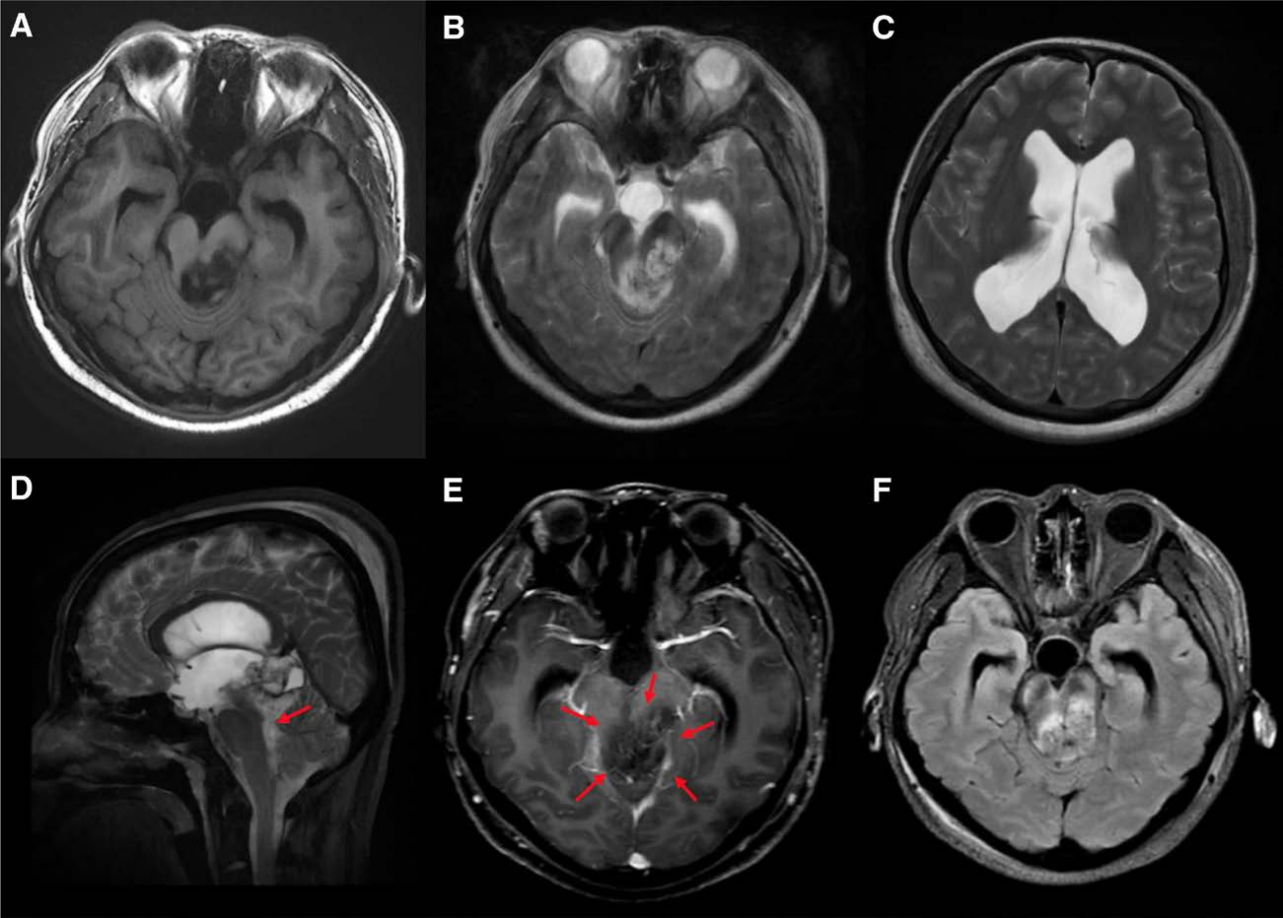
Cranial MRI findings prior to partial surgical resection. Red arrows represented the tumor, measuring approximately 45 × 31 × 52 mm. (A) Axial T1WI: lesion in the brainstem region and left thalamus observed as a heterogeneous hypointense signal. (B, C) Axial T2WI: lesion appeared hyperintense with mild surrounding edema, causing significant compression of the brainstem and narrowing of the cerebral aqueduct, leading to supratentorial ventricular enlargement and hydrocephalus. (D) Sagittal T2WI: lesion extended into the fourth ventricle and compresses the brainstem forward. (E) Axial post-contrast T1WI: a heterogeneous enhancement was seen in the lesion, suggesting active pathology. (F) Axial FLAIR imaging: a heterogeneous mixed signal was observed within the lesion, suggesting a mixture of components of the lesion. FLAIR = fluid-attenuated inversion recovery, MRI = magnetic resonance imaging, T1WI = T1-weighted imaging, T2WI = T2-weighted imaging.

A 35-year-old female presented with sudden syncope that led to urgent hospital admission. The patient’s condition rapidly deteriorated, resulting in impaired consciousness. Cranial CT tomography revealed hydrocephalus, prompting an emergent ventricular puncture and external drainage. Subsequent magnetic resonance imaging (MRI) revealed a mass lesion in the pineal region, extending to the left brainstem and thalamus, measuring approximately 45 × 31 × 52 mm (Fig. 1). The lesion exhibited hypointensity on T1-weighted imaging (T1WI; Fig. 1A) and hyperintensity on T2-weighted imaging (T2WI; Fig. 1B). Compression of the brainstem and narrowing of the cerebral aqueduct were evident, causing supratentorial ventricular enlargement and hydrocephalus (Fig. 1C, D). Post-contrast enhancement was heterogeneous, suggesting active pathology (Fig. 1E). T2 fluid-attenuated inversion recovery imaging demonstrated subcutaneous soft tissue swelling in the bilateral parieto-occipital regions, along with mixed signals within the lesion, indicating the internal heterogeneity of cystic and solid components (Fig. 1F).

**Figure 2.**
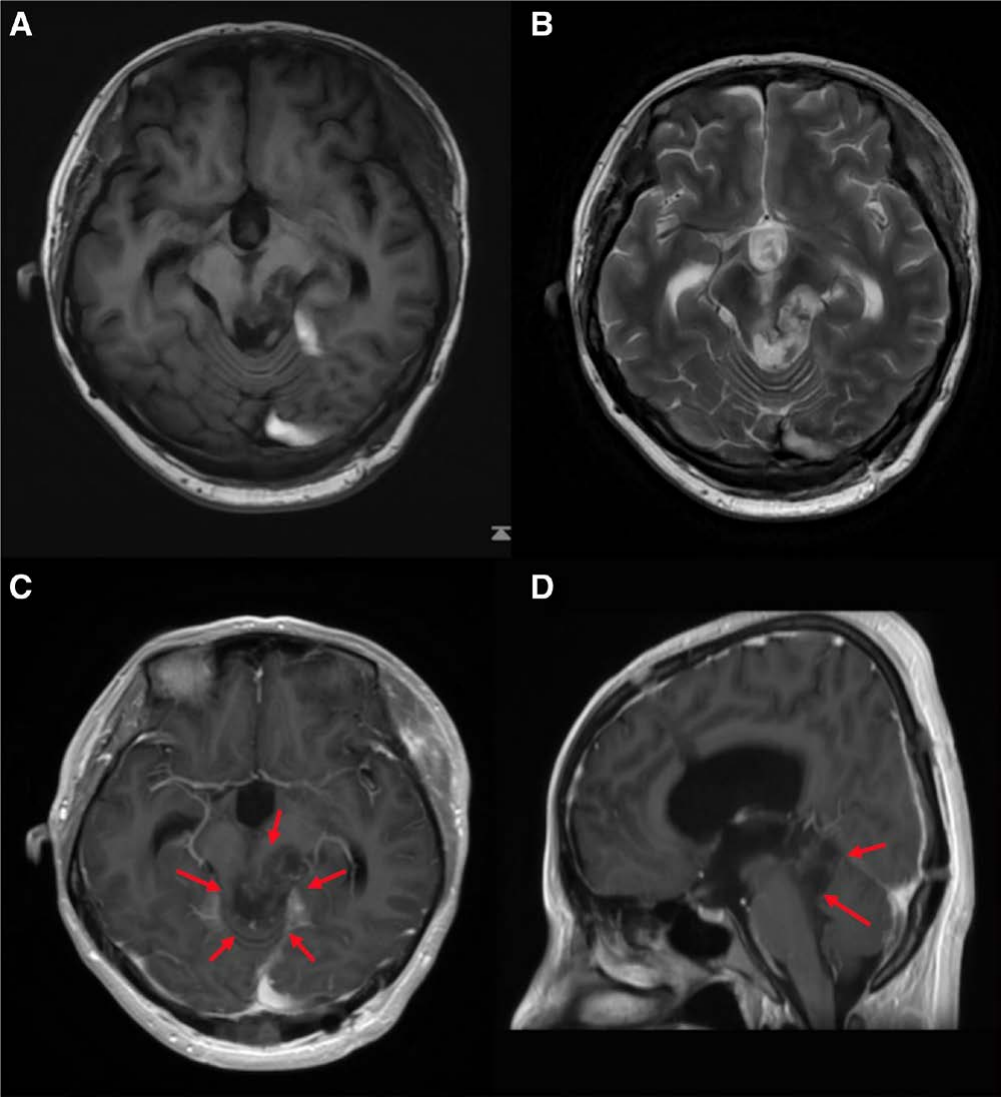
Cranial MRI findings after partial resection and prior to GKRS. Red arrows represented the tumor. A significant portion of the tumor remained in the brainstem and thalamic regions, resulting in compression of the brainstem. (A) Axial T1WI. (B) Axial T2WI. (C) Axial post-contrast T1WI. (D) Sagittal post-contrast T1WI. GKRS = gamma knife radiosurgery, MRI = magnetic resonance imaging, T1WI = T1-weighted imaging, T2WI = T2-weighted imaging.

Since the initial treatments and diagnoses were performed in other hospitals, the medical reports were carefully reviewed. The patient underwent an endoscopic third ventriculostomy and biopsy. Initial pathology revealed a small round cell tumor with localized neuronal rosette formation, suggesting a low-grade glioneuronal mixed tumor, with RGNT considered as a differential diagnosis. Given the critical symptoms and the large size of the tumor, partial surgical resection was performed. However, owing to the involvement of the brainstem and surgical limitations, only a small portion of the pineal tumor was resected, with a significant portion remaining (Fig. 2). Postoperative histopathology confirmed a glioneuronal mixed tumor, with pilocytic astrocytoma-like areas displaying neuronal rosette formations and perivascular pseudorosette patterns. Eosinophilic granular bodies were also observed. Combined with the results of immunohistochemical analysis, glial fibrillary acidic protein (+), neuronal nuclear antigen (+), oligodendrocyte transcription factor 2 (+), S-100 protein (+), and Ki-67 (2%), led to the final diagnosis of RGNT, WHO grade I. Since the patient’s pathology examination was conducted at another institution, we were unable to obtain the original histological slides or HE staining images. However, the pathology report provided by the referring hospital contains a detailed description of the histological findings, which we have included to ensure a comprehensive presentation of the case.

**Figure 3.**
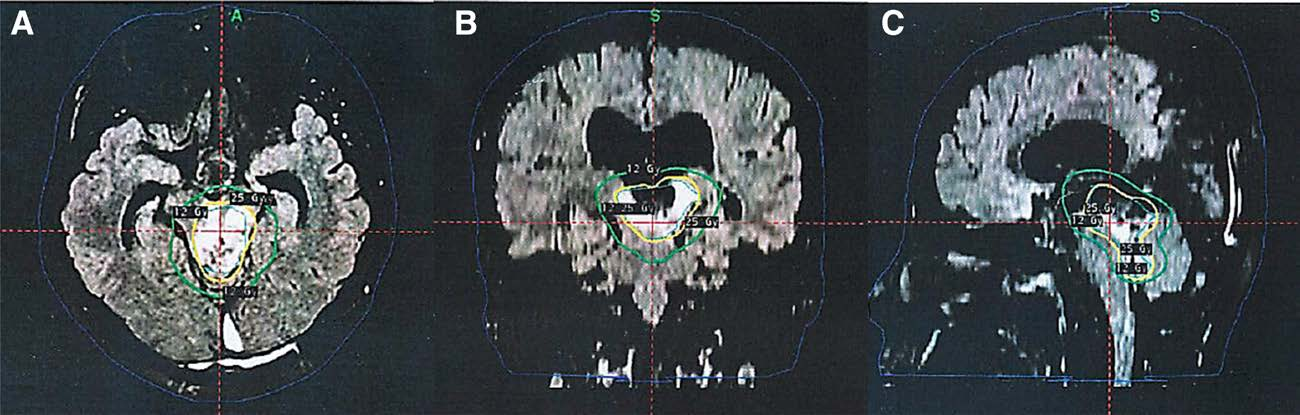
GKRS treatment plan and dose distribution. 25 Gy/5 fractions was performed. The green line represented the 25 Gy isodose line, while the yellow line represented the 12 Gy isodose line. Significant compression of the brainstem was observed at the treatment target, which was enclosed within the higher-dose region. The surrounding tissues, including portions of the ventricles and adjacent structures, were spared from high-dose radiation to minimize adverse effects. (A) Axial dose distribution map. (B) Coronal dose distribution map. (C) Sagittal dose distribution map. GKRS = gamma knife radiosurgery.

To treat the residual tumor in the brainstem and thalamus, where traditional surgery presents significant risks, the patient was referred to our hospital and underwent GKRS, a noninvasive method that provides high precision while minimizing damage to the surrounding tissue. This procedure was performed using the Gamma Knife ICON™ system, which employs a frameless setup. MRI scans were used to create the treatment plan, with the scalp boundaries and target areas delineated using GammaPlan software. A dose of 25 Gy was delivered to the 45% isodose line through 9 shots over 5 fractions (Fig. 3). The tumor volume was 16.57 cm³, with a mean dose of 36.7 Gy. The maximum dose to the tumor center was 55.6 Gy, while the marginal dose was 25 Gy (delivered in 5 fractions, 5 Gy/fraction). For OARs, the brainstem received a dose ranging from 3 to 25 Gy, with a mean dose of 9.8 Gy. The thalamus received a dose ranging from 10 to 25 Gy, with a mean dose of 14.7 Gy. The patient was immobilized using a thermoplastic mask, and cone-beam computed tomography (CBCT) was employed for precise stereotactic localization, with intra-fraction monitoring using an intra-fractional motion management system.

**Figure 4.**
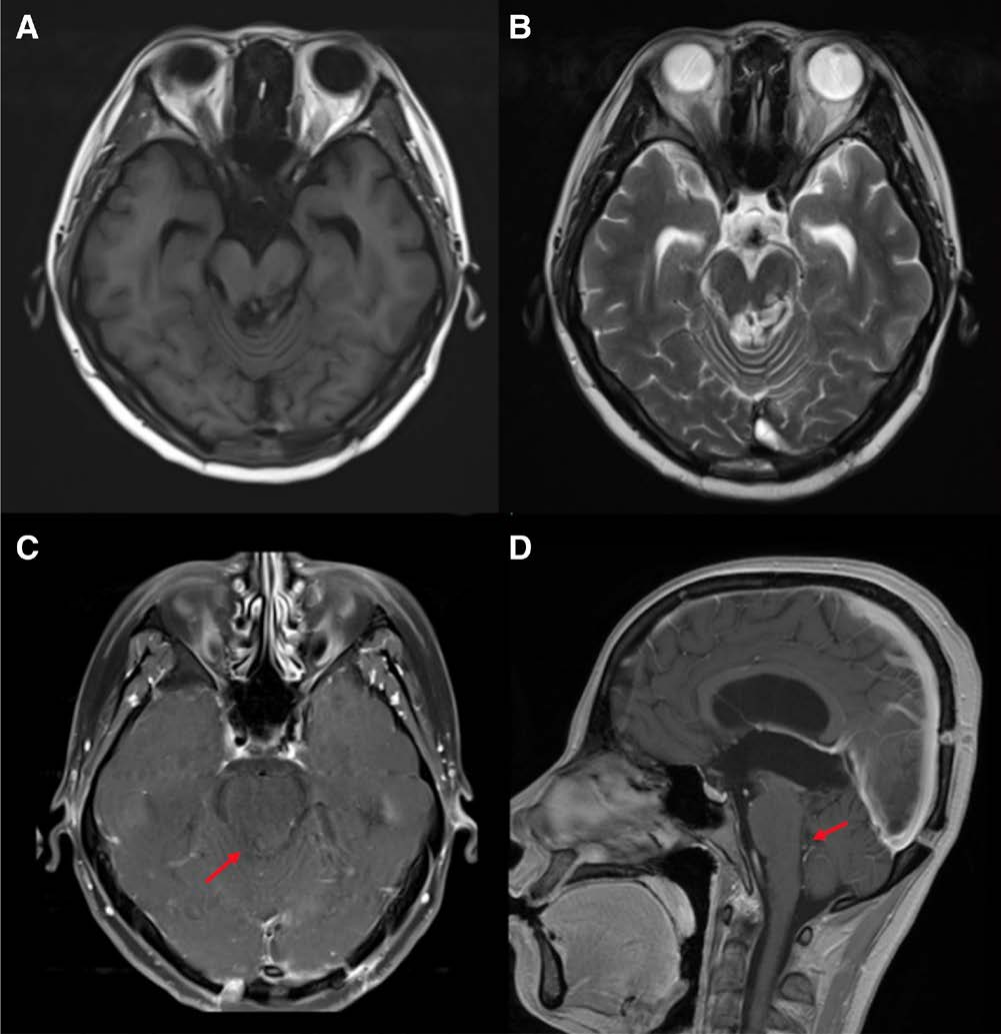
Cranial MRI 3 months post-GKRS treatment. The lesion in the brainstem and thalamic regions was significantly reduced in size, measuring approximately 26 × 13 × 22 mm (indicated by red arrows). (A) Axial T1WI. (B) Axial T2WI. (C) Axial post-contrast T1WI: significant shrinkage of the lesion and no enhancement within the lesion. (D) Sagittal T2WI: lesion in the fourth ventricle almost disappeared, relieving compression of the brainstem. The narrowing of the cerebral aqueduct decreased, and the hydrocephalus improved. GKRS = gamma knife radiosurgery, MRI = magnetic resonance imaging, T1WI = T1-weighted imaging, T2WI = T2-weighted imaging.

The GKRS was successfully and safely completed. Figure 4 illustrates the MRI at 3 months post-GKRS. Tumor volume in the brainstem and thalamic regions was significantly reduced, measuring approximately 26 × 13 × 22 mm. Part of the lesion in the fourth ventricle had almost disappeared, relieving brainstem compression. Post-contrast enhancement showed no enhancement within the tumor. The narrowing of the cerebral aqueduct decreased, and hydrocephalus improved. The patient’s previous neurological symptoms were significantly alleviated, with no side effects such as dizziness, nausea, or vomiting. Since the therapeutic effects of radiation therapy may become more pronounced over an extended period,^[6,7]^ the patient is scheduled for follow-up examinations 1-year post-treatment to evaluate long-term efficacy.

This study was performed in accordance with the principles of the Declaration of Helsinki (2013 version). Ethic approval was granted by the Ethics Committee of Hebei Yizhou Cancer Hospital (Zhuozhou, China).

## 3. Discussion

Given the rarity of RGNTs, there is no global consensus on the optimal treatment strategy. By 2022, only 6 cases of RGNTs in the cerebellar hemisphere had been reported,^[1]^ and fewer than 50 supratentorial RGNT cases were documented by 2023.^[8]^ RGNTs are classified as WHO Grade I tumors, which are typically slow-growing and associated with a favorable prognosis after surgical resection.^[1]^ However, RGNTs can exhibit heterogeneous clinical features, including recurrence, dissemination, and rapid enlargement. Studies have reported a recurrence rate of 14% in partially resected cases.^[9]^ In addition, RGNTs located in critical and high-risk areas, such as the brainstem, still pose significant clinical challenges, because even small tumors can cause severe neurological dysfunction. Therefore, despite the benign nature of the tumor, urgent intervention is required to prevent life-threatening complications and irreversible damage. RGNT is known to cause obstructive hydrocephalus, with headaches and ataxia being the most common clinical manifestations, in addition to different neurological symptoms depending on its location.^[10]^

Surgical resection is typically the treatment option; however, owing to the delicate location of the tumor, complete removal can be challenging.^[11]^ Careful consideration of treatment strategies, including radiotherapy and adjuvant therapies, is required to ensure both tumor control and the preservation of neurological function. Although it is not a standard treatment for RGNT, radiotherapy has shown promising results. Owing to its unique physical dose distribution advantages, proton therapy has been proven to reduce adverse events in normal tissues.^[12,13]^ Yamamoto et al^[14]^ reported no tumor progression within 3 years in a patient with recurrent RGNT of the fourth ventricle treated with a total dose of 50.4 Gy in 28 fractions using proton therapy. Similarly, Ramos et al treated a patient with RGNT using a single 24 Gy fraction of stereotactic radiosurgery. There was no tumor progression, and no adverse reactions were observed within 7 years post-treatment.^[15]^ In this study, immediate intervention was necessary because the patient’s sudden syncope and acute hydrocephalus, which led to impaired consciousness. MRI demonstrated that the tumor’s large volume, deep location, and extension into the brainstem and thalamus limited the extent of resection. The brainstem is densely packed with neural pathways and cranial nerve nuclei, that are essential for survival. Even small errors during surgery can result in severe neurological deficits, including paralysis, breathing difficulties, and death.^[16]^ These constraints underscore the inherent risk of surgical intervention in eloquent brain regions. The decision to perform partial resection was guided by the need to alleviate part of the obstructive hydrocephalus and obtain a pathological diagnosis. The surgery only involved partial resection of the pineal region, leaving substantial tumor remnants in the brainstem and thalamus. Given the urgency and severity of the patient’s symptoms, as well as the infeasibility of enduring prolonged cycles of traditional fractionated radiotherapy or proton therapy, GKRS was deemed the best immediate treatment option to minimize risks and control tumor growth postoperatively.

GKRS has now been established as a noninvasive alternative to microsurgical resection, showing great promise in the treatment of malignant brain tumors as well as benign tumors such as meningiomas, pituitary adenomas, and vestibular schwannomas.^[17]^ Compared to traditional fractionated radiotherapy, GKRS significantly reduces the radiation dose outside the target area, thereby minimizing damage to surrounding neural tissues and reducing adverse effects. Previous models of GKRS typically involved invasive immobilization for single-fraction treatments, leading to complications, such as needle site infections, scar formation, numbness, and pain.^[18]^ However, the patient in this case was treated with the latest GK-ICON^TM^ device, which permits frameless stereotactic radiosurgery technology and hypofractionated treatments. When using the GK-ICON^TM^ device, CBCT was employed to define the stereotactic space, while a thermoplastic mask was used to immobilize the patient. An intra-fractional motion management continuously monitors the patient in real-time to ensure that it remains within the defined stereotactic space. Studies have indicated that the mechanical stability of the CBCT apparatus itself is submillimeter over several months.^[19]^ The thermoplastic mask, a noninvasive immobilization system, does not require anesthetic injections or skin penetration. The incidence of anxiety and pain associated with mask use was lower, resulting in higher patient comfort and acceptance.^[20]^ During the GKRS process in this case, the patient did not show any signs of discomfort or pain, which facilitated smooth implementation and completion of the treatment.

In our case, performing GKRS to alleviate symptoms and reduce tumor size was the most appropriate and least invasive treatment option. The GK-ICON^TM^ device is one of the most advanced technologies in stereotactic radiosurgery that, ensures high levels of comfort and precision during the treatment process. Following GKRS, the patient experienced relief from clinical symptoms without significant acute adverse effects. Despite the promising outcome, this study has several limitations. One major limitation is the unavailability of HE staining and immunohistochemical images, as the pathology examination was conducted at another institution. Due to this, we could not directly present histological images in our report. However, we provided a detailed description of the histopathological findings based on the referring hospital’s pathology report to ensure transparency and completeness. Additionally, the follow-up period after GKRS remains relatively short, and continuous long-term monitoring is required to evaluate the sustained efficacy and associated side effects of GKRS in managing RGNT in high-risk brainstem regions.

## 4. Conclusion

The rarity of RGNT and its invasion into critical brain regions pose significant treatment challenges. This case illustrates the effective application of GKRS in managing rare RGNT in high-risk brainstem regions, highlighting its potential in providing symptom relief in times of crisis and improving patient prognosis. GKRS offers a noninvasive alternative, minimizing risks while controlling tumor growth and alleviating symptoms. The Gamma Knife ICON™ device enhanced patient comfort and treatment precision.

## Author contributions

**Conceptualization:** Shosei Shimizu.

**Data curation:** Dongxue Zhou, Wei Wang, Weiwei Wang.

**Investigation:** Dongxue Zhou, Shuyan Zhang, Chao Liu, Weiwei Wang.

**Methodology:** Yonglong Jin, Shuyan Zhang, Zishen Wang.

**Project administration:** Shosei Shimizu.

**Resources:** Jie Wang, Shuyan Zhang, Chao Liu.

**Software:** Jie Wang, Zishen Wang, Wei Wang.

**Validation:** Yinuo Li.

**Visualization:** Yonglong Jin.

**Writing – original draft:** Zhipeng Shen.

**Writing – review & editing:** Zhipeng Shen, Runzhu Ge, Yinuo Li, Shosei Shimizu.
